# Improvement of analgesic efficacy for total hip arthroplasty by a modified ultrasound‐guided supra‐inguinal fascia iliaca compartment block

**DOI:** 10.1186/s12871-021-01296-8

**Published:** 2021-03-10

**Authors:** Ting Zheng, Bin Hu, Chun-ying Zheng, Feng-yi Huang, Fei Gao, Xiao-chun Zheng

**Affiliations:** 1 The Provincial Clinical Medical College, Fujian Medical University, 134 Dong Street, Fujian 350004 Fuzhou, China; 2 Department of Anesthesiology, Fujian Provincial Hospital, Fuzhou, Fujian China; 3 Fujian Provincial Emergency Center, Fuzhou, Fujian China

**Keywords:** Fascia iliaca, Lumbar plexus block, Magnetic resonance imaging concentrates, Compartment block technique, In‐plane ultrasound‐guided

## Abstract

**Background:**

Fascia iliaca compartment block (FICB) is an anterior approach to the lumbar plexus block and provides the effective adjunctive analgesia for total hip arthroplasty (THA).

**Methods:**

As a case series study, 28 patients (≥ 65 years old) with THA were received a modified in-plane ultrasound-guided supra-inguinal (S-FICB) as an analgesic adjunct to evaluate the analgesic effectiveness and the local anesthetic diffusion with magnetic resonance imaging (MRI). A combination of propofol and sufentanil was administered to conduct target-controlled infusion.

**Results:**

The pain scores were 1 (0–4), 2 (1–5), 3 (1–6) and 3 (1–6) at 4, 8, 12, and 24 h. The cumulative opioids were 8 (8–12), 18 (16–32), 28 (24–54) and 66 (48–104) mg of i.v. morphine equivalents at 4, 8, 12, and 24 h. The patient-controlled analgesia (PCA) times were 0 (0–1), 1 (0–2), 2 (0–5) and 5 (3–8) at 4, 8, 12, and 24 h. In lateral, anterior and medial part of thigh, the sensory blockade in 28 patients was 23 (82 %), 21 (75 %) and 19 (68 %) at 5 min; 28 (100 %) at 10 and 20 min. Motor blockade of femoral nerve (FN) and obturator nerve (ON) was present in 13 (46 %) and 3 (11 %) patients at 5 min, 24 (86 %) and 9 (32 %) at 10 min, 26 (93 %) and 11 (39 %) at 20 min. Injectate permeated to the FN and extended superiorly over the surface of iliac muscle (IM) and pectineus muscle (PM) in all patients.

**Conclusions:**

The modified S-FICB has provided an effective postoperative analgesic adjunct after THA with the satisfactory blockade of femoral (FN), obturator (ON) and sciatic (SN) nerves, especially for ON, when compared with the existing techniques.

## Background

Elderly patients with multiple diseases and comorbidities often suffer hip fractures and necessitating total hip arthroplasty (THA). THA usually results in the severe pain that mainly arises from the site of surgical insertion in the distribution of the lateral femoral cutaneous nerve (LFCN). The subsequent hip joint pain originates from sensory components of the femoral nerves (FN), obturator nerves (ON) and sciatic nerves (SN). FN is the most significant contribution to the hip joint pain. The lumbar plexus block has been used to provides postoperative analgesia for THA, but is limited in the hip surgery because of the high incidence of complications and position requirements. The fascia iliaca compartment block (FICB) is considered as an anterior approach to the lumbar plexus block [1].

**Fig. 1:**
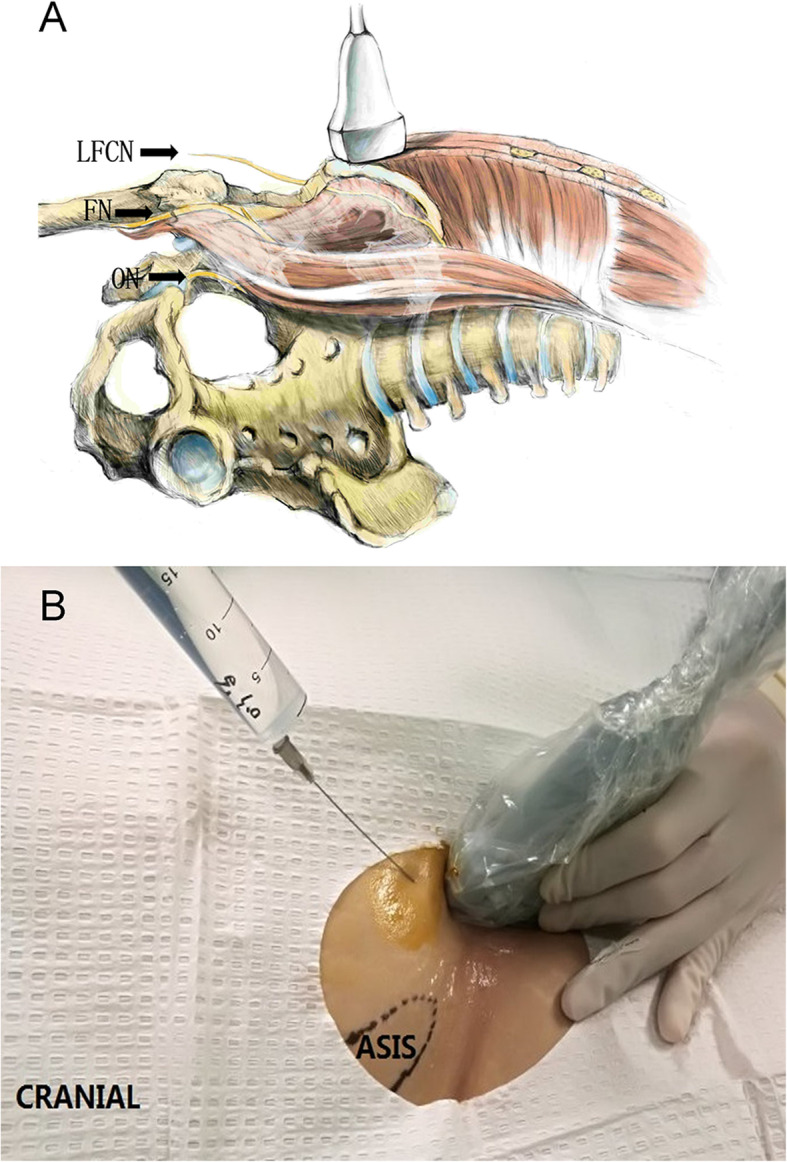
The lateral femoral cutaneous nerve (LFCN) and Transducer position. **a **The course of LFCN. The femoral nerve (FN) and the obturator nerve (ON) in the pelvis (The illustration pictured above is the original work of Ting Zheng. Dr. Zheng holds the right to publish this image.) **b. **Transducer position. Longitudinal orientation adjacent to the anterior superior iliac spine (ASIS) and the position of the puncture needle

The fascia iliaca compartment (FIC) is a potential space between the fascia iliaca and iliacus muscle, which contains FN, LFCN and ON (Fig. 1 A). Dalens et al. [2] have firstly proposed the concept of FICB, because FICB is easy to perform and reaches effective results in blocking three main lumbar plexus nerves supplying the thigh. Since the injection site is distant from major nerves and blood vessels, the FICB technique avoids the complications caused by inadvertent nerve injury or intra-vascular infusion.

The traditional ultrasound-guided FICB is involved in the injection of local anesthetic (LA) inferior to the inguinal ligament, relying on a supra-inguinal spread to block FN and LFCN in the iliac fossa [3, 4]. The approach requires large amounts of LA and yields uncertain effects. In a cadaveric dye-injection study, Hebbard et al. [5] have found that the LFCN is adjacent to FN near the iliac fossa, confirming that an ultrasound-guided supra-inguinal FICB (S-FICB) simultaneously blocks FN and LFCN. Several studies [6–8] have reported that both in-plane and out-plane ultrasound-guided supra-inguinal approaches provide effectively postoperative analgesia and reduce morphine consumption after THA. Vermeylen et al. [1] have demonstrated that S-FICB produces a complete sensory block of the medial, anterior and lateral region of the thigh, and proves LA spreading to the anatomical location of most existing approaches of S-FICB advances the needle in a caudad to cephalad direction. S-FICB and the infra-inguinal FICB (I-FICB) approaches also distribute the injectate detecting by MRI concentrates. Bullock et al. [6] have suggested that the disadvantages in the direction of caudad to cephalad include the need of the ultrasonic anatomical identification and the needle passing many structures to reach target injection site. Therefore, a newly supra-inguinal fascia iliaca approach has been described with a puncturing direction of cephalad to caudad. Bullock have used an out-of-plane technique to puncture the fascia iliaca, which is more operator-dependent because of the difficulty in distinguishing anatomical structures, particularly for practitioners new to the technique. The limitation of Bullock’s study is without the examining the diffusion of ON and LA in MRI.

In this study, we have presented a modified in-plane ultrasound-guided S-FICB technique with placing a probe in the sagittal plane superior to the inguinal ligament, followed by advancing the needle in-plane from cephalad to caudad. The objective of the current case series is to evaluate the analgesic effectiveness of the lumbar plexus nerve block and the distribution of injectate detecting by MRI for the modified S-FICB technique.

## Methods

### The patients

This study was approved by the Ethics Committee of Fujian Provincial Hospital, China (IRB No. K2017-09-020). The current study was a case series, in which all data were prospectively collected to evaluate the efficacy of the modified S-FICB technique. The written informed consents were obtained from all patients for anesthesia, encompassing both general and regional anesthesia. The procedure and possible complications were explained to the patients who agreed to undergo the S-FICB technique as an adjunct anesthesia and analgesic method. Twenty-eight patients with the age of ≥ 65 years undergoing THA diagnosed at the Department of Orthopedics at Fujian Provincial Hospital were scheduled to receive the modified ultrasound-guided S-FICB as an analgesic adjunct. Exclusion criteria were as follows: a history of allergy to local anesthetics; in fictions at the proposed puncture site; a history of coagulopathy; clinical evidence of peripheral neuropathies, cardiac or pulmonary disease; abnormalities of sensory or motor function of the FN, LFCN or ON; refusal to participate in the study.

### The nerve blocks

**Fig. 2:**
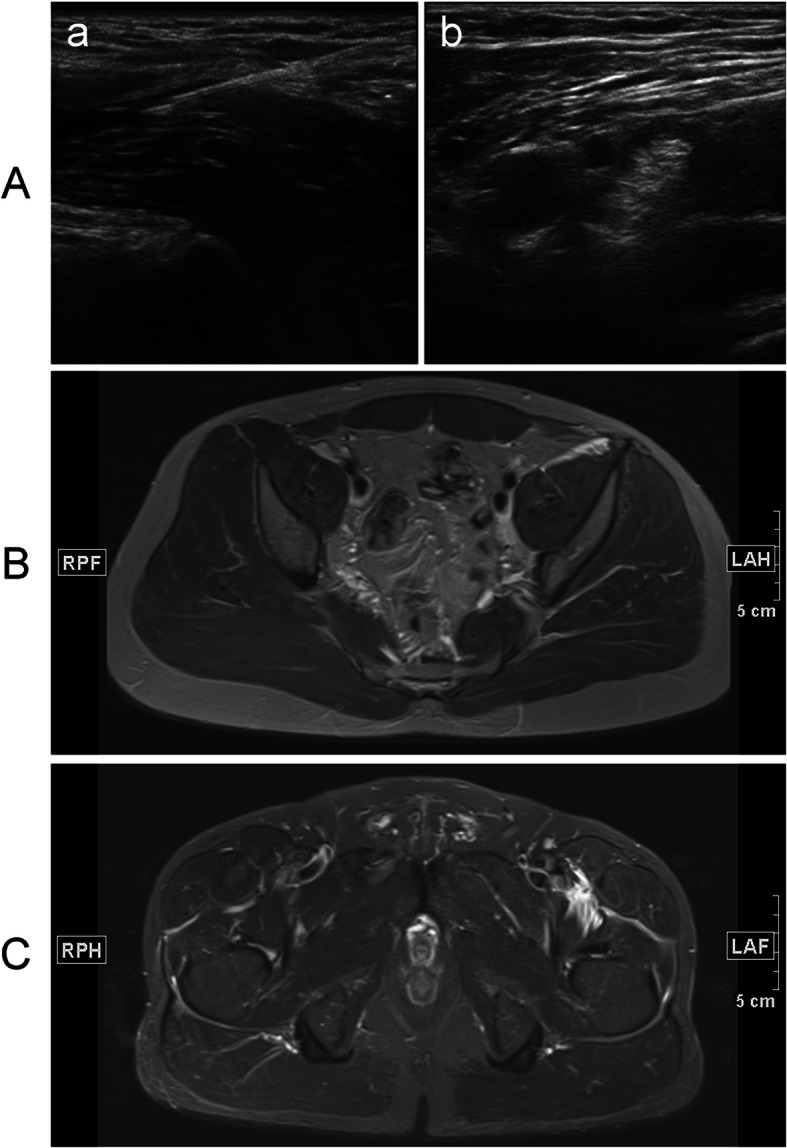
Ultrasound and magnetic resonance images. **a**. Ultrasound images of a novel suprainguinal fascia iliaca compartment *block* (FICB). (**a**) Ultrasound image for identification of the relevant structures for FICB. White arrows, fascia iliaca; *, needle; ASIS, anterior superior iliac spine; SM, sartorius muscle; IOM, internal oblique muscle; IM, iliac muscle; (**b**) local anesthetic around the FN. FI, fascia iliaca; FN, femoral nerve; FA, femoral artery; MED, medial; LAT, lateral. **b**. An axial T2-weighted fat-suppressed magnetic resonance image at the level of the fourth sacral vertebra shows medial spread of injectate (small white arrows) in a plane superficial to the IP muscle and deep to the FA and FV. IP: iliopsoas; ON: obturator nerve; FA: femoral artery; FV: femoral vein. **c**. An axial T2-weighted fat-suppressed magnetic resonance image at the level of the second coccygeal vertebra shows medial spread of injectate (white arrows) in a plane superficial to the IP muscle and diffuses below the pectineus muscle to obturator nerve (☆). PM: pectineus muscle; IP: iliopsoas; OE: obturator externus; OI: obturator internus

Before the procedure, the patient had fasted for 8 h. After entry into the preparation room, the patient was inhaled oxygen, received venous punctures and routinely monitored the blood pressure, electrocardiography, heart rate and pulse. All blocks were preoperatively performed by an experienced anesthesiologist using ultrasound with a 6-13 MHz linear transducer (SonoSite, Inc, Bothell, WA, USA). The anterior superior iliac spine was palpated, and the transducer placed in the sagittal plane to obtain an image of the anterior superior iliac spine and identify the iliac muscle by sliding the probe medially. The probe was then adjusted to identify the ultrasound anatomy, including subcutaneous tissue, internal oblique muscle, sartorius muscle, fascia iliaca, and iliac muscle. A needle (21-gauge and 100-mm block) was advanced in an in-plane fashion to puncture the fascia iliaca (Fig. 1B). When the needle tip was just below the fascia iliaca and the pumping without gas and blood, 5 ml of saline was injected to confirm the tip location. Once the proper position was confirmed, 30 mL of 0.3 % ropivacaine was incrementally injected superficial to the iliac muscle and deep to expand the fascia iliaca through the water separation technique (Fig. 2 a, b). When the injection was finished, the transducer was medially translated toward FN. Therefore, LA was identified spreading around the transducer (Fig. 2 a, b).

### The MRI and anesthesia

After the sensory and motor blockade were checked, the patient was evaluated using MRI. The inferior and superior limits of image acquisition were the lesser trochanter of the femur and the second lumbar vertebrae, respectively. General anesthesia was administered after MRI examination. A combination of propofol and sufentanil was administered with a dual-channel target control system to conduct target-controlled infusion. The target concentration of propofol was set at 3 µg/ml (plasma target control, Marsh mode) and sufentanil at 0.2 ng/ml (effector target control, Gepts mode) to maintain the bispectral index (BIS) between 40 and 60. Once the mean blood pressure (MBP) rose more than 20 % of baseline during the surgery, the target concentration of sufentanil was increased by 0.05 ng/ml every 5 min until the MBP returned to the basal level. Ephedrine was used to maintain hemodynamic stability when MBP decreased more than 20 % of baseline. The target sufentanil concentration was decreased to 0.08 ng/ml and the propofol concentration was decreased to maintain BIS around 60 at 30 min prior to the end of the surgery, and the propofol was stopped at the end of the surgery.

### Postoperative analgesia

For the postoperative anesthesia, a patient-controlled intravenous analgesia (PCIA) system with sufentanil (bolus-only mode; background dose, 2 ml/h; bolus, 2 ml; lockout, 15 min; maximal dose, 30 ml/4 h) was started on arrival at the post-anesthesia care unit (PACU). The button of patient-controlled analgesia (PCA) was pressed by the patient while the visual analogue scale (VAS) score was ≥ 4 points. If the pain intensity did not reduce within 10–20 min, tramadol was administered for supplementary analgesia by the ward doctor.

### Study outcomes

The outcomes were the sufentanil, tramadol and cumulative opioids 4, 8, 12 and 24 h postoperatively. Opioid doses were converted to intravenous morphine equivalents for normalization [9]. Pain scores at rest were recorded by independent investigators 4, 8, 12, and 24 h postoperatively using a VAS ruler on a scale from 0 to 100 mm, with 0 representing no pain and 100 corresponding to the worst pain imaginable [10]. Sensory and motor blockade were evaluated 5, 10 and 20 min after ropivacaine administration, using loss of perception to cool in the lateral, anterior and medial part of the thigh (corresponding to LFCN, FN and ON nerve sensory distributions, respectively) [11]. Motor strength was graded between 0 and 5 according to the Medical Research Council (MRC) scale. Motor blockade was evaluated by testing knee extension (corresponding to FN) and thigh adduction (corresponding to ON) [12]. The result of LA diffusion was detected by MRI. The scanning parameter of MRI was according to the former [12].

### Statistical analysis

Statistical analysis was performed using SPSS 20.0 software. Data are presented as mean ± SD in cases of the normally distributed data or median (range) otherwise. Standard hypothesis tests (2-sided *t* test or Mann-Whitney *U* test) were performed to analyze baseline characteristics and outcome parameters. Categorical data were assessed using frequency tables and χ^2^ or Fisher exact test. The primary hypothesis was performed at a 5 % significance level.

## Results

The age range of patients in this case series was between 65 and 78 years old. In 28 patients, 17 (61 %) were males while 11 (39 %) females; 15 (54 %) had the body weight less than 60 kg and 13 (46 %) more than 60 kg.

By using Mann Whyitney-u test [6], the pain scores were 1 (0–4), 2 (1–5), 3 (1–6) and 3 (1–6) at 4, 8, 12, and 24 h, respectively (Table 1).

**Table 1:** Pain scores

Hours	4	8	12	24
VAS	1 (0–4)	2 (1–5)	3 (1–6)	3 (1–6)
Sufentanil (µg)	8 (8–12)	18 (16–22)	28 (24–34)	56 (48–64)
Tramadol (mg)	0 (0–0)	0 (0–50)	0 (0-100)	50 (0-200)
Cumulative opioids (mg)	8 (8–12)	18 (16–32)	28 (24–54)	66 (48–104)
PCA time	0 (0–1)	1 (0–2)	2 (0–5)	5 (3–8)

Note: *VAS* visual analogue scale; *PCA* principal components analysis

Using Mann Whitney-u test. Score 1 = median, 0 = minimum, 4 = maximum pain values

The cumulative opioids were 8 (8–12), 18 (16–32), 28 (24–54) and 66 (48–104) mg of IV morphine equivalents at 4, 8, 12, and 24 h. The PCA times were 0 (0–1), 1 (0–2), 2 (0–5) and 5 (3–8) at 4, 8, 12, and 24 h, respectively (Table 1).

In lateral, anterior and medial part of thigh, the sensory blockade in 28 patients was 23 (82 %), 21 (75 %) and 19 (68 %) at 5 min; 28 (100 %), 28 (100 %) and 28 (100 %) at 10 and 20 min, respectively, in all thigh parts (Table 2).

**Table 2:** Sensory blockade after FICB at different time intervals

	Present n (%)	Absent n (%)
Lateral thigh		
5 min	23 (82 %)	5 (18 %)
10 min	28 (100 %)	0 (0 %)
20 min	28 (100 %)	0 (0 %)
Anterior thigh		
5 min	21 (75 %)	7 (25 %)
10 min	28 (100 %)	0 (0 %)
20 min	28 (100 %)	0 (0 %)
Medial thigh		
5 min	19 (68 %)	9 (32 %)
10 min	28 (100 %)	0 (0 %)
20 min	28 (100 %)	0 (0 %)

Note: *FICB* fascia iliaca compartment block

Motor blockade of FN and ON in 28 patients was 13 (46 %) and 3 (11 %) at 5 min; 24 (86 %) and 9 (32 %) at 10 min; 26 (93 %) and 11 (39 %) at 20 min, respectively (Table 3).

**Table 3:** Motor blockade after FICB at different time intervals

Time after injection	Knee extension n(%)	Hip adduction n(%)
5 min 10 min 20 min	13/28(46 %) 24/28(86 %) 26/28(93 %)	3/28(11 %) 9/28(32 %) 11/28(39 %)

Injectate permeated to the FN and extended superiorly over the surface of the IM and PM in all patients (Fig. 2b). In all 28 patients, superior distribution was limited to the level of the fourth sacral vertebra. In 12 of 28 patients, injectate permeated below the pectineus muscle to obturator nerve at the level of the second coccygeal vertebra (Fig. 2 c).

None of the patients developed any local anesthetic systemic toxicity or showed prolonged sensory, motor deficits, symptoms suggestive of neurologic injury or other complications.

## Discussion

In the case series study, we have included 28 patients undergoing THA to evaluate the safety and effectiveness of the modified S-FICB for adjunctive analgesia in THA and observe the distribution of LA using MRI. To evaluate a clinical block of FN and LFCN, a sensory assessment of the anterior and lateral part of the thigh is adequate. The assessment of ON block is difficult, because of an inconsistent cutaneous distribution of ON [1, 2]. Therefore, the testing motor blockade of thigh adduction has been used to evaluate ON and the knee extension to the blockade of FN. Our results have demonstrated that the modified S-FICB technique completely blocks the medial, anterior and lateral thigh. Opioid consumption is decreased during the block (within ~ 12 h) with a subsequent increase after the block resolution (~ 12–24 h). Cumulative opioids and PCA times are more objective measurement of pain compared to subjective pain scores, suggesting that the block is functional. The pain scores have not changed because of the supplementary analgesic and the complex innervation of the hip joint.

In our procedure, we did not use the caudal to cranial approach but have used the cranial to caudal approach to avoid the groin area that is too close to the perineum area and easily to be infected. The only artery above the iliac fascia is the deep iliac circumflex artery, just like the puncture of the iliac fascia under the ligament. The iliopsoas muscle under the fascia is strong. The fascia level is easily to identify. Therefore, we have kept the puncture needle in the ultrasound beam, which is without the puncture risk. The cranial to caudal approach has been confirmed to be effective and can be used as a research basis for the later catheterization [5, 13].

The hip joint is dominated by multiple nerves, including FN, LFCN, ON, and the articular branches of the sciatic nerve [13]. Therefore, regional techniques to block the lumbar plexus, such as FICB and psoas compartment block, are preferred for postoperative analgesia in the hip surgery. FICB is relatively easier to perform with a lower risk in blocking the branches of the lumbar plexus, such as spinal and epidural anesthesia, nerve injury and retroperitoneal hemorrhage [1]. Several studies have also reported that FICB provides an analgesic effect after the hip surgery using I-FICB or S-FICB technique [6, 11, 14].

Cutaneous analgesia for most hip incisions is mainly through the lateral femoral cutaneous nerve (LFCN), which supplies sensation to the lateral aspect of the thigh. LFCN has an inconsistent course below the inguinal ligament with variable branching [15], but also shows a more consistent course above the inguinal ligament. Therefore, a nerve block with LA injection above the inguinal ligament yields the better anesthesia than the I-FICB technique doe, because I-FICB is involved in the injection of the LA inferior to the inguinal ligament and does not reliably block these nerves. By using I-FICB technique, Shariat et al. [16] have failed to provide the adequate analgesia and could not decrease the opioid consumption in patients after THA. The incidence of successful FN, LFCN and ON block is only 38 %, 31 % and 25 %, respectively. By I-FICB, all three nerves (FN, LFCN and ON) are simultaneously blocked in only 2 of 16 patients. Therefore, more research on S-FICB has been carried out in the recent years.

There are several reports about using the imaging technique to analyze the distribution pattern of LA. Marhofer et al. [17] have evaluated the spread of LA after I-FICB using the sensory blockade and MRI testing, concluding that LA acts on the anterior branch of the obturator nerve by slightly spreading in medial direction and extends to the posterior branch. In contrast, Swenson et al. [12] have used MRI to evaluate the LA distribution after I-FICB and shown that no patients demonstrate the decrease in the hip adductor strength and no evidence of the ON block at any level on MRI. Vermeylen et al. [1] have performed a randomized controlled trial in healthy volunteers using MRI to compare the effects of I-FICB and S-FICB. In 80 % of the volunteers, LA is present in the anatomical location of ON after S-FICB compared with 10 % after I-FICB. The results have not been unexpected because I-FICB fails to demonstrate a reduction in the morphine consumption or pain intensity. We have demonstrated the decrease in the hip adductor strength in 12 of 28 patients and a satisfactory sensory blockage in the medial part of the thigh. Moreover, an axial T2-weighted fat-suppressed MRI at the level of the second coccygeal vertebra has shown the medial spread of injectate in a plane superficial to the iliopsoas muscle and diffuses below the pectineus muscle. The anterior branch of obturator nerve descends in the front of the obturator externus and passes between the adductor brevis (deep) and the pectineus of adductor longus (superficial). The spread of LA to the deep surface of the pectineus muscle suggests the presence of obturator nerve blockade. These analyses might explain the absent sensory in the medial thigh and the reason of the significant weakness of adductor strength in the patients. The case series have firstly demonstrated that not only injectate permeates to ON, but also leads to the adductor strength weakness.

We have presented a modified S-FICB technique placing a probe in the sagittal plane superior to the inguinal ligament to obtain a satisfactory ultrasound image, followed by advancing the needle in-plane from cephalad to caudad. The advantage of the modified S-FICB technique is that the sonographic anatomy is clearly identified from superficial to deep. Bullock et al. [6] have described the injection of a local anesthetic below the fascia iliaca using the supra-inguinal fascia iliaca technique. In contrast to our modified S-FICB technique, they have used an out-of-plane technique to puncture the fascia iliaca, which is more operator-dependent because of the difficulty in distinguishing anatomical structures, particularly for practitioners who are new to the technique. Different form the technique by Bullock et al., in our modified S-FICB technique with an in-plane approach, the tip can be easily and safely reached to puncture the fascia iliaca. The site of the inserting needle is far away from the surgical incision to avoid affecting sterilization and operating procedures. We have also used the ON examine to avoid a limitation of Bullock’s study, in which the ON examine has not been used.

There are some limitations in the present study. First, the designed case series is only in a single-center clinical study with addition of a potential bias element. Before this study, we have performed more than 200 blocks in patients with THA using this S-FICB approach and obtained satisfactory blocking without complications. To reduce the potential bias, a multicenter study is needed to verify the effectiveness of the technique. Second, we have not designed a randomized controlled study to compare with other techniques. Although our technique has yielded satisfactory effects and confirmed the LA diffusion, we cannot declare that our technique is superior to others. Therefore, we need a randomized controlled study. Finally, we have not observed the decrease in the hip adductor strength in all patients, although we have demonstrated that LA has satisfactorily spread to FN and LFCN and ON has approximately blocked in half of 28 patients with THA. We intend to increase LA capacity or concentration in the future studies.

## Conclusions

In summary, the modified S-FICB has provided an effective postoperative analgesic adjunct to patients with total hip arthroplasty (THA) with the satisfactory blockade of femoral (FN), obturator (ON) and sciatic (SN) nerves, especially for ON, when compared with the existing techniques.

## Data Availability

All data generated or analyzed during this study are included in this published article.

## Abbreviations

THA: Total hip arthroplasty
LFCN: Lateral femoral cutaneous nerve
FN: Femoral nerve
ON: Obturator nerve
SN: Sciatic nerves
FICB: Fascia iliaca compartment block
FIC: Fascia iliaca compartment
LA: Local anesthetic
S-FICB: Supra-inguinal FICB
MRI: Magnetic resonance imaging
I-FICB: Infra-inguinal FICB
BIS: Bispectral index
MBP: Mean blood pressure
PCIA: Patient-controlled intravenous analgesia
IV: Intravenous
PACU: Post-anesthesia care unit
