# Correction to: Improvement of analgesic efficacy for total hip arthroplasty by a modified ultrasound-guided supra-inguinal fascia iliaca compartment block

**DOI:** 10.1186/s12871-021-01314-9

**Published:** 2021-03-31

**Authors:** Ting Zheng, Bin Hu, Chun-ying Zheng, Feng-yi Huang, Fei Gao, Xiao-chun Zheng

**Affiliations:** 1 Shengli Clinical Medical College of Fujian Medical University, Fuzhou, Fujian China; 2 Department of Anaesthesiology, Fujian Provincial Hospital, Fuzhou, China; 3 Fujian Emergency Medical Center, Fuzhou, China

**Correction to: BMC Anesthesiol 21, 75 (2021)**

**https://doi.org/10.1186/s12871-021-01296-8**

Following publication of the original article [1], the authors reported an error in: the authors’ institution and the Fig. 2 has a minor adjustment. Because Our author’s institution has recently made a change, so I must to replace all the authors’ institution as follow:

- 1 Shengli Clinical Medical College of Fujian Medical University, Fuzhou, Fujian, China
- 2 Department of Anaesthesiology, Fujian Provincial Hospital, Fuzhou, China
- 3 Fujian Emergency Medical Center, Fuzhou, China

**Fig. 2:**
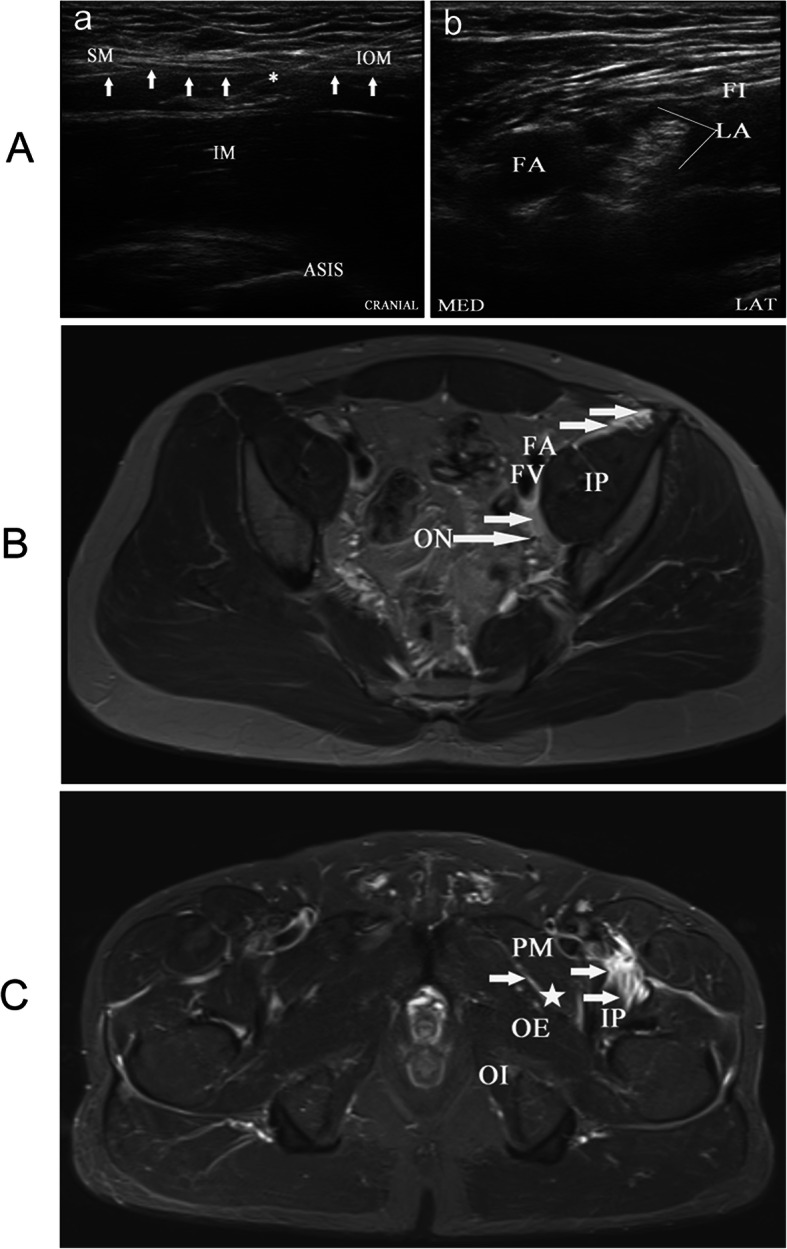
Ultrasound and magnetic resonance images. **a**. Ultrasound images of a novel suprainguinal fascia iliaca compartment block (FICB). (**a**) Ultrasound image for identification of the relevant structures for FICB. White arrows, fascia iliaca; *, needle; ASIS, anterior superior iliac spine; SM, sartorius muscle; IOM, internal oblique muscle; IM, iliac muscle; (**b**) local anesthetic around the FN. FI, fascia iliaca; FN, femoral nerve; FA, femoral artery; MED, medial; LAT, lateral. **b**. An axial T2- weighted fat-suppressed magnetic resonance image at the level of the fourth sacral vertebra shows medial spread of injectate (small white arrows) in a plane superficial to the IP muscle and deep to the FA and FV. IP: iliopsoas; ON: obturator nerve; FA: femoral artery; FV: femoral vein. **c**. An axial T2-weighted fatsuppressed magnetic resonance image at the level of the second coccygeal vertebra shows medial spread of injectate (white arrows) in a plane superficial to the IP muscle and diffuses below the pectineus muscle to obturator nerve (☆). PM: pectineus muscle; IP: iliopsoas; OE: obturator externus; OI: obturator internus
